# Fellowships, Grants, & Awards

**Published:** 2007-03

## Social and Cultural Dimensions of Health (R01)

The ultimate goal of this program announcement is to encourage the development of health research that integrates knowledge from the biomedical and social sciences. This involves the further development of health-related social science research relevant to the missions of the NIH Institutes and Centers (ICs) and the development of multi- or interdisciplinary research that blends the theories and approaches of the social and biomedical sciences. Within the broad spectrum of research identified in this announcement, applicants are encouraged (but are not required) to employ multiple (i.e., biological, behavioral, and/or social) levels of analysis.

This announcement invites applications to 1) elucidate basic social and cultural constructs and processes used in health research; 2) clarify social and cultural factors in the etiology and consequences of health and illness; 3) link basic research to practice for improving prevention, treatment, health services, and dissemination; and 4) explore ethical issues in social and cultural research related to health.

This program announcement is a conversion and revision of PA-02-043 and is based on recommendations submitted to the NIH in conjunction with the conference titled “Toward Higher Levels of Analysis: Progress and Promise in Research on Social and Cultural Dimensions of Health,” 27–28 June 2000, Bethesda, MD. For a summary of the conference, see http://obssr.od.nih.gov/Documents/Conferences_And_Workshops/HigherLevels_Final.PDF. Also see “Social Science and Health Research: Growth at the NIH,” American Journal of Public Health, 94(1) Jan. 2004:22–28. You may request these publications from the Office of Behavioral and Social Sciences Research, Office of the Director, National Institutes of Health, Bethesda, MD 20892.

Social scientists have made significant strides in shedding light on the basic social and cultural structures and processes that influence health. Social and cultural factors influence health by affecting exposure and vulnerability to disease, risk-taking behaviors, the effectiveness of health promotion efforts, and access to, availability of, and quality of health care. Social and cultural factors also play a role in shaping perceptions of and responses to health problems and the impact of poor health on individuals lives and well-being. In addition, such factors contribute to understanding societal and population processes such as current and changing rates of morbidity, survival, and mortality.

Numerous reports from the Institute of Medicine and National Research Council have pointed to the importance of social and cultural factors for health and the opportunities for improving health through a better understanding of mechanisms linking the social and cultural environment to specific health outcomes. To realize these opportunities, social science research related to health must be further developed and ultimately integrated into interdisciplinary, multilevel studies of health. Linking research from the macrosocietal levels, through behavioral and psychological levels, to the biology of disease will provide the integrative health research necessary to fully understand health and illness.

This program announcement invites applications for research on the social and cultural dimensions of health in five areas: 1) basic social and cultural constructs and processes used in health research; 2) etiology of health and illness; 3) consequences of poor health for individuals and social groups; 4) linking science to practice to improve prevention, treatment, health services, and dissemination; 5) ethical issues in social and cultural research.

The goal of this announcement is to encourage further development of health-related social sciences research relevant to the missions of the ICs. These missions encompass a broad range of scientific questions related to the health and well-being of our nation's people. Information about the specific missions of the ICs participating in this program announcement is posted at http://www.nih.gov/icd/.

### Basic Constructs and Processes

Advances in social science research on health depend on a foundation of basic theory and knowledge that describes social structures, the dynamics of social and cultural processes, and the ways in which individuals are located in and interact with the environment, social structures, and cultural factors. Several key sociodemographic constructs, including race, ethnicity, gender, age, and socioeconomic status, are widely used in studies of the etiology of health and disease and in research that describes and monitors the distribution of disease across social categories, geographic areas, and time. However, the meanings of such constructs depend on their cultural, geographical, and historical context, and their utility in health research depends on their use in ways that are theoretically and historically grounded. In addition, the concept of culture requires careful theoretical grounding in health studies. Most social scientists agree that the concept of culture is complex and implies a dynamic and ever-changing process.

This program announcement encourages research on basic social and cultural constructs and processes in the following areas:

#### Social Stratification and Inequalities

Research is needed to explore the implications of different conceptualizations and measurements of social stratification systems and processes, such as socioeconomic status (SES) and social class, age, gender, and race/ethnicity for understanding health at the individual and higher levels of aggregation (e.g., community). Research to improve the monitoring and understanding of inequalities in health and disease among diverse groups, and the implications for monitoring of strategies used to measure basic constructs such as socioeconomic status and social class, age, gender, race, and ethnicity.

The National Research Council‘s study of Critical Perspectives on Racial and Ethnic Differences in Health and Late Life discusses several issues related to racial and ethnic inequalities in health, including the nature of racial and ethnic differences, an outline of causal pathways implicated in health disparities, and a research agenda in the field of racial and ethnic differences in health. (See N.B. Anderson, R.A. Bulatao, and B. Cohen, Editors, Panel on Race, Ethnicity, and Health in Later Life, National Research Council, *Critical Perspectives on Racial and Ethnic Differences in Health in Late Life*, Washington, DC:National Academies Press, 2004.)

#### Social Integration

Research is required to clarify the social, cultural, and economic factors that influence the social integration of individuals and the social cohesion of groups, including the causal dynamics of social networks.

#### Culture

Studies are necessary to improve the conceptualization and operationalization of culture as well as of social and cultural change in health research. Efforts are needed to identify those definitions and dimensions of cultural phenomena and intracultural and intercultural variation and change that are most useful in understanding health, and the mechanisms through which cultural phenomena influence health.

### Etiology

Social science research on the etiology of health and illness recognizes that health may be affected by a diverse set of mechanisms operating among and within social structures existing at different levels. At the highest levels are structures and processes that involve and affect populations broadly: government, media, economic systems, social stratification, political processes and policy making, and broadly held cultural values and practices. Some of these processes also operate in communities and neighborhoods, in social institutions (e.g., schools, churches, and businesses), and in social or professional organizations. However, at these levels processes contributing to social cohesion, social support, social control, social and cultural conflict, and the development and enforcement of social and cultural norms play a larger role. In families and small groups, interpersonal processes such as conflict and support, socialization, and sharing of resources play a dominant role.

A valuable contribution of the social sciences is to understand health and disease not solely as an individual biological problem, but as a social phenomenon associated with social ties and other forms of social influences. From this perspective, research must address how mechanisms that link social and cultural phenomena to health operate within and emerge from specific social contexts. Social contexts provide the stage for social and cultural factors to influence health, and the characteristics of social context directly affect social and cultural processes.

This program announcement encourages research on topics and questions such as the following:

#### Overarching Issues

Research is needed to improve understanding of how macrolevel (societal) factors, such as social policies, structures, and cultural norms, are linked to microlevel (individual) factors, such as a person's behaviors, and ultimately to health. What are the causal pathways that lead from the sociocultural environment to general vulnerability to disease and disease-specific outcomes? Research that integrates theories and methods from the social and biomedical sciences is particularly encouraged to address these questions.

#### Interpersonal, Social, and Cultural Factors

Essential are studies of the implications for health of the characteristics and content of network ties and of how individuals and groups organize themselves into networks and other social arrangements, including the mechanisms through which social integration/ cohesion, social influence, and other social processes affect the health of individuals and contribute to health disparities. More research is needed on cultural processes and belief systems (such as religion or the nature of health/disease), at the individual, family, community, and institutional levels, and their relationships to health, including recovery from disease and addiction, with particular attention to potential mediating mechanisms (e.g., socially determined patterns of stress and coping with stress).

#### Social Contexts

Research is considered necessary on the role of social contexts (e.g., family and households, religious institutions, workplaces, schools, health care organizations and systems, neighborhoods, and communities, geographic location, residential segregation, legal and administrative policies, communication environments) in mediating or moderating sociocultural influences on health of individuals. Studies are required to conceptualize and measure social contexts in order to specify which particular aspects of social context are relevant to health and the mechanisms through which they operate. This includes research on how health policies impact on diverse populations, such as those defined by immigration status, gender, race/ethnicity, sexual orientation, or age, and on the pathways through which social policies (such as gun control, urban renewal, welfare reform, and taxes on alcohol and tobacco products) affect the health of diverse populations.

### Consequences of Poor Health

Connections among health, functional capacity, and productivity are complex and difficult to disentangle, but empirical research is emerging that addresses the consequences of poor health for economic well-being at the individual, family, and population levels. Understanding the consequences of health and illness is important to the mission of the NIH. First, health disparities among groups varying in SES result in part from the reciprocal influence of SES on health and health on SES. The nature of these feedbacks needs to be fully understood if we are to understand the mechanisms underlying health disparities. Second, the value of investment in improving health can be only partially understood by focusing on health outcomes alone. For example, improvements in quality of life resulting from social, economic, and cultural change at both the individual and societal level are an important part of the picture.

This program announcement encourages research on the consequences of poor health, such as the following:

#### Self Care

Research is desired on self care or self-regulation (including the choice of complementary or alternative medical practices) as a response to illness and in the management of health conditions, considering the influence of social, cultural, and economic factors on the adoption and consequences of this strategy.

#### Coping Strategies

Required are investigations of the coping strategies people use to adapt to illness and disability, the influence of social, cultural, and economic factors on these strategies, and the impact of these strategies on health and well-being at the individual, family, and community level. Research on the consequences of death and dying for the health and well-being of the deceased's relatives and friends as well as on the coping strategies people use to adapt to illness, disability, and death of a relative or close friend.

#### Social Stigma

Needed are studies of stigma across physical and mental health conditions (including addictions), care settings, outcomes and groups, including research on the social and cultural origins of stigmatization of illnesses. What are the implications of stigma for access to care and treatment? How does stigma affect outcomes across health conditions?

#### Impact of Health on Society

Research is necessary to examine how the health of individuals impacts upon macrolevel processes and systems is also needed. How does the health of individual members of a group (e.g., family, household, firm) affect the composition and functioning of the group? Also of interest is research on the influence of poor health on economic performance of organizations and societies. (For example, see International Studies of Health and Economic Development, NIH Guide to Grants and Contracts, 30 May 2000; http://grants.nih.gov/grants/guide/rfa-files/RFA-TW-01-001.html)

### Linking Science to Practice to Improve Prevention, Treatment, Health Services, and Dissemination

The social sciences are important in efforts to prevent and treat illness and to promote health. Research in the social sciences can pinpoint environmental contexts, social relationships, interpersonal processes, and cultural factors that lead people to engage in healthy behaviors, seek health services before disease symptoms worsen, and participate with medical professionals in treating illness. The incorporation of social science research and theory into prevention, treatment, service, and health-promotion programs is likely to result in more effective interventions. In addition, research on the dissemination and translation of social science research findings can ensure that investments in basic research have their maximum impact on health.

This program announcement encourages social sciences research on prevention, treatment, health services, health promotion, and information and program dissemination in the following areas:

#### Prevention

Greater theoretical development and conceptual work is needed in the field of prevention, including clarifying the concepts of risk and protection and their meanings within distinct populations, defining the distinctions between health promotion and disease prevention, and promoting generalizability of theoretical frameworks. Research is desirable to design, implement, and evaluate interventions based upon the theories, concepts, and methods identified earlier in this announcement (e.g., social networks, social contexts, cultural beliefs).

#### Treatment and Management of Disease

Research is needed on cultural competence at multiple levels, including health systems, agencies and providers, with an emphasis on primary care and mental health settings. Also, research is essential to define what constitutes “culturally competent care,” develop and test different models (best practices) of culturally competent care, and test models in randomized controlled trials. Research is desired that explores the interface between traditional/ alternative and allopathic/Western medicine and health maintenance practices and identifies the circumstances under which either or both function more effectively.

#### Services

Research on the development, dissemination, and accessibility of new therapies, technologies and services, such as retrovirals and antipsychotics. How do social and cultural factors affect these processes and what impact do they have on services and treatment? How do social, cultural, economic, and policy mechanisms influence equitable access to health care and the quality of care received?

#### Dissemination and Adoption

Research is essential on the processes through which social and behavioral interventions are incorporated into general practice. What accounts for success or failure (i.e., adoption versus nonadoption)? How does this differ from the adoption of biomedical treatments and interventions? Systematic research is required on methods to increase the adoption of tested and effective preventive interventions, treatment models, and service delivery strategies. Also increased research is needed that will allow rigorous comparisons of the effects of alternative methods of diffusion and dissemination.

### Ethical Issues in Social and Cultural Research

The development of new and more complex research methods in the social sciences, combined with dramatic advances in computing power, complicates standard ethical concerns of confidentiality, privacy, and consent. Higher levels of analysis imply analysis of data at the group, institution, or community level, raising the prospects of consent at these levels and how such consent might be obtained. Sensitivity exists not only at the individual level but also for the groups and institutions with which individuals affiliate.

This program announcement encourages social sciences research on ethical issues in the following areas:

Ethical issues arising from research that links the individual to groups, organizations, neighborhoods, or communities.

Threats to confidentiality of data collected in multilevel studies by advancing statistical methods for masking or altering individual data and studying how such procedures impinge on the ability to conduct valid analyses.

Unintended consequences of research aimed at understanding variation among individuals and among groups. How can we avoid overemphasizing individual and group differences and thereby reinforcing existing patterns of stratification in health care or other areas?

Community consultation in research projects involving identified population groups. How can individual informed consent best be accomplished in this setting?

This Funding Opportunity Announcement (FOA) will use the Research Project Grant (R01) award mechanism.

The applicant will be solely responsible for planning, directing, and executing the proposed project.

This FOA uses “Just-in-Time” information concepts. It also uses the modular as well as the non-modular budget formats (see http://grants.nih.gov/grants/funding/modular/modular.htm).

Specifically, if you are a U.S. organization and are submitting an application with direct costs in each year of $250,000 or less (excluding consortium Facilities and Administrative [F&A] costs), use the PHS398 Modular Budget component provided in the SF424 (R&R) Application Package and SF424 (R&R) Application Guide (see specifically Section 5.4, “Modular Budget Component,” of the Application Guide).

U.S. applicants requesting more than $250,000 in annual direct costs and all foreign applicants must complete and submit budget requests using the Research & Related Budget component found in the application package for this FOA. See NOT-OD-06-096

Applicants must download the SF424 (R&R) application forms and the SF424 (R&R) Application Guide for this FOA through Grants.gov/Apply.

Note: Only the forms package directly attached to a specific FOA can be used. You will not be able to use any other SF424 (R&R) forms (e.g., sample forms, forms from another FOA), although some of the "Attachment" files may be useable for more than one FOA.

For further assistance, contact GrantsInfo, 301-435-0714, (telecommunications for the hearing impaired: TTY 301-451-0088), or by e-mail: GrantsInfo@nih.gov.

The application submission dates are available at http://grants.nih.gov/grants/funding/submission-schedule.htm.

The complete version of this PA is available at http://grants.nih.gov/grants/guide/pa-files/PA-07-045.html.

Contacts: The complete list of agency contacts is available at http://grants.nih.gov/grants/guide/pa-files/PA-07-045.html.

Reference: PA-07-045.

